# Periodontal health in a longitudinal French cohort of people with Parkinson’s disease

**DOI:** 10.1038/s41598-026-55641-3

**Published:** 2026-06-05

**Authors:** J. Samot, E. Courtin, D. Guehl, N. Damon-Perriere, O. Branchard, M. Saint-Jean, V. Chuy, B. Ella, M. C. Badet

**Affiliations:** 1 Department of Oral Surgery, CHU Bordeaux, Bordeaux, F-33000 France; 2 Department of Clinical Neurophysiology, Bordeaux University Hospital, Bordeaux, France; 3 University of Bordeaux, 146 rue Leo Saignat, Bordeaux, F-33000 France; 4 University of Bordeaux, INRAE, Bordeaux INP, Bordeaux Sciences Agro, UMR 1366, OENO, ISVV, Villenave d’Ornon, F-33140 France; 5 Department of Neurosurgery B, Bordeaux University Hospital, Pellegrin Hospital, Bordeaux, F-33000 France; 6 University of Bordeaux, INSERM, BPH, Bordeaux, U1219, F-33000 France; 7 Department of Dentistry and Oral Health, CHU Bordeaux, Bordeaux, F-33000 France; 8 University of Bordeaux, Institute of Neurodegenerative Disorders, UMR 5293, Bordeaux, F-33000, France

**Keywords:** Oral hygiene, Dental plaque, Periodontal diseases, Microbiota, Parkinson’s disease, Diseases, Health care, Medical research, Microbiology, Neurology

## Abstract

Motor impairment associated with Parkinson’s disease (PD) can hinder daily oral hygiene. This study aimed to assess the microbiological and clinical evolution of periodontal health in people with PD. This prospective cohort study included participants with PD. Clinical oral health data and microbiological samples were collected at baseline and after a follow-up period (median 7 months [range: 3–21]). Forty people with PD were recruited (35% female; median [Q1–Q3] age: 65.3 [57.9–72.8] years). The participants had good hygiene habits; however, no significant changes were observed in clinical parameters, including an increase in the proportion with a ‘good’ plaque index (+ 21 points, *p* = 0.239) and the reduction in the frequency of periodontal pockets > 6 mm (from 20% to 3%, *p* = 0.131). In contrast, using mixed-effects models accounting for follow-up time and intra-individual variability, a significant reduction in *Treponema denticola* bacterial load was observed (93% reduction in CFU/mL; 95% CI: 69%–98%; adjusted *p* = 0.012). Clinical oral hygiene parameters remained stable, while a significant change was observed in one microbiological parameter. These findings require confirmation in larger studies to better define oral care support for PD patients.

**Trial registration**: ClinicalTrials.gov Identifier: NCT03827551, registered on January 31, 2019, (https://clinicaltrials.gov/study/NCT03827551).

## Introduction

Parkinson’s disease (PD) is a neurodegenerative disease characterized by motor and non-motor symptoms. PD affects approximately 1% of people over the age of 60 worldwide (between 6 and 7 million people in total)^1,2^. It is the second most common neurodegenerative disorder in the world after Alzheimer’s disease and the fastest growing neurological disorder worldwide^3,4^.

The diminished motor skills and impaired fine hand movements accompanying PD complicate daily oral hygiene procedures, and studies have shown reduced oral health and oral hygiene care in patients with PD^5–8^. Consequently, individuals with PD have an overall higher prevalence of oral diseases and orofacial dysfunctions, including higher prevalences of caries, gingivitis, periodontitis, and tooth loss^8,9^.

Periodontal disease has a complex etiology involving microbial, inflammatory, and genetic processes and ranges clinically from simple, fully reversible gingivitis to complex forms of periodontitis. The latter, which affect the deep structures of the periodontium and destroy connective tissue and bone, result in loss of attachment, which can lead to tooth mobility and eventual tooth loss. Periodontal disease is associated with gingival recession and bleeding linked to the presence of dental plaque and calculus^10–13^. The progression of periodontal lesions increases with the severity of PD^14^. At a microbiological level, the extent of periodontal damage seems to be directly related to the total bacterial load and to the presence of bacterial species such as *Porphyromonas gingivalis*, *Aggregatibacter actinomycetemcomitans*, or *Treponema denticola*^15,16^.

A recent review by Murcia-Flores et al. (2025) did not identify any longitudinal studies evaluating changes in periodontal microbiota in individuals with PD, highlighting a major gap in the literature regarding temporal microbial dynamics in this population^17^. Furthermore, no studies in France have examined the oral health of people with PD, although such knowledge could enable patients to receive appropriate advice and improve their oral care to enhance their quality of life. Importantly, the absence of repeated quantitative assessments of periodontal pathogens limits our understanding of how dysbiosis evolves over time in people with PD. Given the association between specific bacterial species and periodontal dysbiosis and disease progression, longitudinal microbiological assessment may provide important insights into periodontal health dynamics and support the development of targeted preventive or therapeutic strategies.

In this context, our primary objective was to describe the microbiological evolution of oral health in individuals with PD, using quantitative measurements of periodontal pathogens collected at two time points during their medical follow‑up. As a secondary objective, we evaluated the clinical evolution of periodontal status over the same period.

## Results

### Demographic data

This study included 40 patients with PD (65% male; median age 65.3 [range 39.4–81.1] years) (Table 1). All participants were community-dwelling. At baseline, the median disease duration was 14.5 years. Median UPDRS Part III and Hoehn and Yahr scores were 14.5 and 2, respectively, indicating a good level of autonomy. Two participants had type 2 diabetes. Regarding smoking status, six participants were former smokers (cessation 4–30 years prior to inclusion), and one was an active smoker with a 35 pack-year history. The follow-up visit was disrupted by the COVID-19 crisis. The median time to follow-up was 7 [range 3–21] months. Thirty-five patients completed a follow-up visit, and five withdrew from the study for health reasons (e.g., broken femur, fatigue, deterioration in general condition).

**Table 1:** Participant characteristics at baseline.

Characteristics at baseline	Total (*N* = 40)
Female	14 (35)
Age (years)	65.3 [57.9–72.8]
UPDRS part III Score, 2 m.d.	14.5 [10.0-23.5]
Hoehn and Yahr Score, 2 m.d.	2.0 [2.0–3.0]
Duration of the disease (years), 2 m.d.	14.5 [9.0–20.0]

Values in N (%) for the qualitative variable and in median [1st quartile – 3rd quartile] for quantitative variables.

m.d.: missing data; UPDRS: Unified Parkinson’s Disease Rating Scale.

### Levodopa equivalent daily dose (LEDD)

At baseline, the mean ± SD LEDD was 784.2 ± 638.7 mg/d, and a non-significant decrease was observed at follow-up (778 ± 597.4 mg/d, *p* = 0.96). One patient had missing data at both baseline and follow-up.

### Microbiological evolution

A major decrease in the median bacterial load between baseline and follow-up was observed for *T*. *denticola*, dropping from a pathogenic threshold to a physiological threshold (Table 2). The estimated effect was − 2.66 (95% CI: -4.14, -1.17; non-adjusted *p* = 0.001; adjusted *p* = 0.012). After back-transformation from the natural log scale, this corresponds to an estimated 93% [98%, 69%] reduction in CFU/mL. For *C. rectus*, a decrease was also observed, with an estimated effect of -1.42 (95% CI: -2.79, -0.06; 76% [94%, 6%] reduction; non-adjusted *p* = 0.048). However, this association did not remain statistically significant after correction for multiple testing (adjusted *p* = 0.242). For *A*. *actinomycetemcomitans*, the bacterial load was zero at both baseline and follow-up, indicating quantities below the PCR detection threshold. For the other bacteria and for total bacterial load, quantities remained similar between baseline and follow-up and no significant time-related change was observed.

**Table 2:** Median concentrations (ln-transformed) of periodontal bacteria at baseline and follow‑up, and time‑related change estimated with linear mixed‑effects models (*N* = 40).

Periodontal bacteria	Manufacturer pathogenicity threshold	Baseline Median [Q1-Q3]	Follow-up visit Median [Q1-Q3]	Effect estimate on log scale [95% CI]	% change in CFU/mL (back‑transformed)	*p*-value	Adjusted *p*-value
Total flora	Healthy periodontium < 16.1 Gingivitis < 18.4 Periodontitis ≤ 18.4	21.7 [20.7–22.5]	21.4 [20.8–22.6]	-0.21 [-0.74, 0.32]	-19 [-52, 38]	0.445	0.556
*Aggregatibacter actinomycetemcomitans*	6.9	0.0 [0.0–0.0]	0.0 [0.0–0.0]	0.11 [-0.39, 0.61]	12 [-32, 84]	0.666	0.666
*Porphyromonas gingivalis*	11.5	11.1 [0.0-14.3]	10.4 [0.0–12.0]	-1.16 [-2.87, 0.56]	-69 [-94, 75]	0.196	0.379
*Tannerella forsythia*	11.5	12.9 [11.4–13.9]	11.8 [9.8–14.4]	-0.89 [-2.59, 0.81]	-59 [-92, 125]	0.314	0.488
*Treponema denticola*	11.5	13.6 [10.9–15.8]	11.1 [8.7–13.8]	-2.66 [-4.14, -1.17]	-93 [-98, -69]	**0.001**	**0.012**
*Prevotella intermedia*	11.5	16.2 [13.7–17.5]	16.5 [13.9–17.4]	-0.42 [-1.74, 0.90]	-34 [-82, 146]	0.541	0.601
*Parvimonas micra*	13.8	14.1 [12.5–15.3]	14.2 [11.8–15.3]	-1.02 [-2.34, 0.30]	-64 [-90, 35]	0.137	0.363
*Fusobacterium nucleatum*	16.1	15.9 [15.3–17.0]	15.9 [14.1–17.0]	-0.87 [-2.02, 0.29]	-58 [-87, 34]	0.145	0.363
*Campylobacter rectus*	13.8	14.6 [13.2–15.5]	13.2 [11.7–15.1]	-1.42 [-2.79, -0.06]	-76 [-94, -6]	**0.048**	0.242
*Eikenella corrodens*	16.1	13.2 [11.5–14.4]	11.4 [8.2–14.2]	-1.24 [-3.23, 0.74]	-71 [-96, 110]	0.227	0.379

Missing data: 2 (5%) at baseline; 5 (13%) at follow‑up for all variables except 6 (15%) for *A. actinomycetemcomitans*.

Mixed models included a random intercept for participants and were adjusted for individual follow‑up delay.

P‑values were corrected for multiple comparisons using the Benjamini–Hochberg approach.

CFU: colony-forming unit; CI: confidence interval; mL: millimeter; Q: quartile.

In the secondary analyses, the odds of having *T. denticola* counts above the pathogenicity threshold at follow-up were 73% lower than at baseline (OR = 0.27, 95% CI 0.08–0.89; non-adjusted *p* = 0.031; Table 3), after accounting for baseline risk and follow-up delay. However, this association did not remain statistically significant after correction for multiple testing (adjusted *p* = 0.248). For all other bacteria, the odds of exceeding the pathogenicity threshold did not differ significantly between baseline and follow-up. More than 95% of patients had total bacterial counts consistent with periodontitis at baseline, compared with 85% at follow-up.

**Table 3:** Prevalence of participants with periodontal bacteria above pathogenicity thresholds at baseline and follow-up, and time‑related change estimated with logistic models (*N* = 40).

Periodontal bacteria	Baseline *N* (%)	Follow-up visit *N* (%)	OR [95% CI]	*p*-value	Adjusted *p*-value
Total flora *					
Healthy periodontium	0	0			
Gingivitis	0	1 (3)			
Periodontitis	38 (95)	34 (85)			
*Aggregatibacter actinomycetemcomitans* * ≥10^0.3^ (log10 CFU/mL)	3 (8)	3 (8)			
*Porphyromonas gingivalis* ^a^ ≥10^0.5^ (log10 CFU/mL)	17 (42)	13 (32)	0.64 [0.21, 1.98]	0.437	0.699
*Tannerella forsythia* ^b^ ≥10^0.5^ (log10 CFU/mL)	28 (70)	19 (48)	0.42 [0.16, 1.13]	0.086	0.344
*Treponema denticola* ^a^ ≥10^0.5^ (log10 CFU/mL)	27 (68)	16 (40)	0.27 [0.08, 0.89]	**0.031**	0.248
*Prevotella intermedia* * ≥10^0.5^ (log10 CFU/mL)	34 (85)	31 (78)			
*Parvimonas micra* ^b^ ≥10^0.6^ (log10 CFU/mL)	23 (58)	18 (45)	0.69 [0.27, 1.74]	0.427	0.699
*Fusobacterium nucleatum* ^b^ ≥10^0.7^ (log10 CFU/mL)	18 (45)	17 (42)	1.04 [0.41, 2.62]	0.931	0.995
*Campylobacter rectus* ^a^ ≥10^0.6^ (log10 CFU/mL)	24 (60)	16 (40)	0.45 [0.16, 1.28]	0.134	0.357
*Eikenella corrodens* ^b^ ≥10^0.7^ (log10 CFU/mL)	2 (5)	3 (8)	0.96 [0.07, 12.79]	0.975	0.995

* Estimation could not be obtained due to insufficient variability in the outcome and model instability.

Missing data: 2 (5%) at baseline; 5 (13%) at follow‑up for all variables except 6 (15%) for *A. actinomycetemcomitans*.

^a^ Fixed-effects models.

^b^ Mixed-effects models included a random intercept for participants and were adjusted for individual follow‑up delay.

P‑values were corrected for multiple comparisons using the Benjamini–Hochberg approach.

Pathogenicity thresholds are in log10 CFU/mL

CI: confidence interval; OR: odds ratio.

### Changes in periodontal clinical status

In total, 37.5% of the participants reported no dental examinations in the previous year (Table 4). Among those with regular dental appointments, 16% reported three or more dental visits in the previous year. All reevaluated patients reported at least one dental visit since baseline.

**Table 4:** Changes in periodontal clinical status between baseline and follow‑up.

Characteristics	Baseline (*N* = 40)	Follow-up visit (*N* = 35)	p*
Dental visit in the past year	25 (62)		
Number of visits to the dentist in the past year (for those with ≥ 1 visit)			n.a.
1	16 (40)		
2	5 (13)		
≥ 3	4 (10)		
Number of visits to the dentist since baseline, 3 m.d.			n.a.
None		10 (29)	
1		19 (54)	
2		3 (9)	
Toothbrushing frequency, 1 m.d.			0.134
< 2 times/day	12 (30)	7 (20)	
≥ 2 times/day	27 (68)	27 (77)	
Plaque index, 1 m.d. at follow-up			0.239
Good	20 (50)	25 (71)	
Fair	20 (50)	9 (26)	
Calculus index, 1 m.d. at follow-up			0.248
Good	35 (88)	31 (89)	
Fair	5 (13)	3 (9)	
OHI-S, 1 m.d. at follow-up			0.121
Good	27 (68)	30 (86)	
Fair	13 (33)	4 (11)	
CPITN status			0.131
Healthy periodontium	10 (25)	8 (23)	
Bleeding during or after periodontal probing	3 (8)	6 (17)	
Presence of calculus	4 (10)	10 (29)	
Periodontal pocket 4–5 mm	15 (38)	10 (29)	
Periodontal pocket > 6 mm	8 (20)	1 (3)	
CPITN status for posterior teeth			0.091
Healthy periodontium	11 (28)	12 (34)	
Bleeding during or after periodontal probing	7 (18)	10 (29)	
Presence of calculus	2 (5)	3 (9)	
Periodontal pocket 4–5 mm	13 (33)	9 (26)	
Periodontal pocket > 6 mm	7 (18)	1 (3)	

Values in N (%).

m.d.: missing data; CPITN: Community Periodontal Index for Treatment Needs; n.a.: not applicable; OHI-S: Simplified Oral Hygiene Index.

* McNemar’s Chi-squared test with continuity correction.

Most participants reported brushing their teeth twice a day at baseline, and a non-significant increase in the frequency was observed at the follow-up visit (68% vs. 77%, *p* = 0.134). There was also a non-significant reduction in the plaque index between baseline and the follow-up visit, whereas the calculus index remained stable. Therefore, the OHI-S increased, although not significantly (68% vs. 86% with good oral hygiene, *p* = 0.121).

Regarding periodontal status, there was a decrease in the frequency of patients with healthy periodontium, but also decrease in the frequency of patients with periodontal pockets, with 20% at baseline of patients having periodontal pockets > 6 mm versus 3% follow-up (overall, *p* = 0.131 for CPITN status at baseline vs. follow-up). These changes did not reach statistical significance. A similar pattern was observed for posterior teeth.

## Discussion

In this study, the participants tended to maintain good oral hygiene habits, as reflected in both toothbrushing frequency and plaque levels at baseline and follow-up. Over time, CPITN findings suggested a possible reduction in the proportion of sites associated with deeper periodontal pockets, particularly those exceeding 6 mm. Additionally, there was a significant decrease in *T*. *denticola* counts, along with reductions in the total bacterial load and the load of six other bacterial species. However, despite these observed trends, more than 7 in 10 patients continued to have bacterial counts consistent with periodontitis throughout the study.

The toothbrushing frequency observed in this study was similar to that reported in other studies for populations with PD^5,18,19^. According to Einarsdottir et al., who investigated individuals with PD in Iceland, these levels were even higher than those observed in a control population, although the difference was not significant^5^. Notably, these results were obtained despite the absence of a specific dental care policy for people with PD in Iceland at the time.

Although our study participants appeared to follow toothbrushing recommendations, many still had plaque accumulation, suggesting ineffective brushing techniques. At follow-up, after all participants reported having consulted a dentist, plaque levels were reduced, possibly reflecting a positive impact of these consultations or even participation in the study itself. Similar reductions in plaque levels have been reported by Baram et al., who observed a significant reduction in the plaque index 2 months after an intervention in two groups of approximately 15 patients^20^.

The assessment of calculus presence is more complex due to the use of two different indices in this study: the calculus index integrated into the OHI-S, and the CPITN. Based on the OHI-S alone, the presence of calculus was similar between baseline and follow-up, whereas participants’ oral hygiene showed a reduction in plaque levels. When assessed using the CPITN, calculus appeared more prevalent among patients who attended the follow-up visit. Nevertheless, the CPITN analysis also revealed a non-significant decrease in periodontal pockets exceeding 4 mm in depth, particularly in posterior teeth. This trend may reflect a potential shift in periodontal condition, despite the persistence of calculus. Although plaque levels decreased over time, the continued presence of substantial calculus can be explained by the lack of scaling between baseline and follow-up. Since calculus forms from mineralized plaque that has not been entirely removed, its persistence is expected. Scaling is a professional procedure requiring a dentist, whereas plaque removal can be performed at home.

Consistent with these findings, the need for periodontal care was high at enrollment due to the presence of deep periodontal pockets, but showed a tendency to improve over time. Overall, these data suggest that despite multiple risk factors for poor oral health, people with PD seem to actively engage in maintaining good oral hygiene. However, evidence on the effectiveness of interventions targeting oral health in this population remains limited, with only one randomized controlled trial reported to date^20,21^.

Among studies assessing PPD in individuals with PD, few have used the CPITN, and those that have tend to report only a global score, which limits insight by failing to differentiate between individual CPITN components^22,23^. However, other studies have highlighted increased pocket depth in people with PD. Cicciu et al. reported that 75.8% of cases presented with pocket depths greater than 5 mm, whereas Hanaoka and Kashihara found that 99% of individuals with PD had pockets exceeding 4 mm, compared to 44% in their control group^6,10^. In our study, deep periodontal pockets were prevalent in the posterior dental region. When combined with tooth mobility, this can cause pain and significantly impair masticatory function. Moreover, increased pocket depth in molars ultimately worsens their long-term prognosis for retention in the dental arch^24^.

Interestingly, despite persistent calculus, exploratory CPITN findings suggested fewer sites classified with deeper periodontal pockets over time. Previous studies have shown that gingival health can show signs of recovery even in the presence of calculus, provided that plaque levels are reduced^25^. This may highlight the potential role of microbial control in managing periodontal disease in this population. In our study, the choice of CPITN to evaluate periodontal health was driven by feasibility and ethical considerations in a population with PD, in whom motor impairment, fatigue, and reduced tolerance for prolonged clinical examinations may limit the applicability of full-mouth periodontal recordings. Although full-mouth PPD and CAL assessments are recommended for detailed periodontal characterization in epidemiological research^26^, such approaches may not always be appropriate in vulnerable populations. CPITN therefore represents a pragmatic screening tool in this context, but its use may limit the detection of site-specific changes and the interpretation of clinical–microbiological associations.

The microbiological data further emphasize a precarious periodontal state, with five of the nine bacteria studied having median values above defined pathogenicity thresholds and a bacterial load incompatible with periodontal health. Limited data exist on the composition and abundance of periodontal disease-associated bacterial flora in individuals with PD. In a cohort of about 20 patients, Yay et al. observed that the subgingival microbiome associated with periodontitis was altered in people with PD. In deep periodontal pockets, *P*. *micra*, *C*. *rectus*, and *T*. *denticola*, among others, were detected more frequently in individuals with PD compared to those with periodontitis in the general population^27^. Despite follow-up and a trend toward fewer deep periodontal pockets, many patients retained high bacterial loads, with only one exhibiting a total bacterial load compatible with periodontal health. This observation is consistent with findings by He et al., who demonstrated that PPD correlates poorly with the total bacterial load, but is more strongly associated with specific pathogens such as *P*. *gingivalis*, *T*. *forsythia*, and *T*. *denticola*^28^. In our cohort, only *T. denticola* load significantly decreased over time. This reduction may reflect a trend toward improved plaque levels, but could also result from factors that have not yet been fully identified. Some authors have suggested that catecholamines, including dopaminergic agents, influence bacterial load and ultimately dental health^29,30^. However, such an effect is unlikely in our study, as the mean LEDD remained relatively stable over time.

Addressing the bacterial drivers of inflammation in periodontitis is crucial, as numerous studies suggest that periodontal lesions contribute to systemic inflammation, potentially exacerbating neuroinflammation^31–33^. In addition, characterizing differences in these microbial communities is important, as regional variation in the distribution of bacterial species associated with severe periodontal disease has been reported^34^. Further studies incorporating more extensive microbiological data, including broader microbiome profiling, are therefore needed.

This study has several limitations that should be considered when interpreting the findings. The single-center design and relatively small sample size may limit generalizability and reduce statistical power to detect subtle clinical changes, particularly for outcomes showing non-significant trends. In addition, small sample sizes may reduce the stability of effect estimates and increase uncertainty. The absence of a control group precludes causal inference; observed changes over time may therefore reflect temporal trends, regression to the mean, or residual confounding. In particular, adjustment for potential confounders such as smoking status and type 2 diabetes was not feasible given their low prevalence in this cohort ; therefore, residual confounding cannot be excluded. As participants were community-dwelling and relatively autonomous, the findings may not be directly applicable to individuals with more advanced disease or those living in institutional settings. From a microbiological perspective, the analysis was restricted to a targeted panel of periodontal pathogens, which allowed quantitative assessment of key species using validated methods but does not capture the full complexity of the oral microbiome. Periodontal disease is a polymicrobial, dysbiotic condition in which key pathogens such as *T. denticola* and *P. gingivalis* represent only a small fraction of the microbial community, and thus single-species changes may not reflect broader ecological shifts^35^. Accordingly, although a significant reduction in *T. denticola* was observed, its persistence in a context of overall periodontitis-associated profiles suggests limited translation into clinical improvement. Finally, the variable follow-up duration, largely driven by COVID-19-related disruptions, introduced heterogeneity in assessment timing and may have contributed to variability in outcomes. Pandemic-related factors may also have influenced oral health behaviors and access to care. Despite these limitations, the longitudinal design remains a strength, allowing within-subject assessment of clinical and microbiological parameters over time using validated indices and biological measures, thereby reducing information bias.

Our findings highlight a positive trend in oral hygiene among individuals with PD included in this study. Although these promising results require validation, they are an important step toward strengthening oral care support for people with PD. However, both our study and previous ones focused primarily on patients with relatively mild PD. Future research should extend these findings by including individuals with more advanced disease. Finally, this study, along with recent research on the oral health of people with PD, contributes to the development of tailored recommendations for their multidisciplinary management. By expanding the literature on oral health in PD, our findings reinforce existing recommendations, such as those summarized by Martimbianco et al., which outline key strategies to improve the quality of life of individuals with Parkinson’s disease^36^.

## Methods

### Study design and population

This work was part of PARKIDENT, a prospective cohort study designed as an exploratory longitudinal pilot study on the oral health of patients with PD (ClinicalTrials.gov identifier NCT03827551; https://clinicaltrials.gov/study/NCT03827551). The research protocol was reviewed and approved by a French regional ethics committee, the Comité de Protection des Personnes (CPP) Sud-Est III (approval number EudraCT N° 2018-A02773-52). This study adhered to the STROBE guidelines and all procedures were conducted in accordance with applicable regulations and ethical standards, including the principles of the Declaration of Helsinki.

All participants gave informed consent. Participants were recruited between April 2019 and September 2020 at the Institute of Neurodegenerative Diseases of Bordeaux (France) and were all community-dwelling individuals. At the time of study design, no prior data were available to support a formal sample size calculation; the sample size was therefore determined based on the anticipated recruitment capacity within the predefined 12-month inclusion period. Participants were included according to the following inclusion criteria : have PD, older than 18 years of age, able to give informed consent, and affiliated with the national health insurance. Participants were not included if they had another condition that could affect the oral microbiome (antibiotic or antiseptic mouthwash use in the past month), if they were pregnant or wore a subtotal (fewer than six teeth with no molars or incisors) or total dental prosthesis. Including follow-up visits, data collection continued until April 2021.

### Data collection

At baseline, gender and age were recorded, along with a clinical assessment of PD. This assessment included the Movement Disorder Society–Unified Parkinson’s Disease Rating Scale (MDS-UPDRS) for motor PD symptoms (UPDRS part III), the Hoehn and Yahr scale for PD staging, and the duration of the disease since age at diagnosis^37,38^. To address the issue of different PD drug regimens, conversion factors for antiparkinsonian drugs that have been calculated to provide a total levodopa equivalent daily dose (LEDD) were used^39^.

Oral health data were collected at baseline and at reassessment at least 3 months later by two dentists, after a calibration phase conducted on ten patients not included in this study. A 6-month follow-up was initially planned; however, the actual follow-up duration varied due to disruptions related to the COVID-19 pandemic. The number of dental visits in the past year for baseline, or since baseline for the follow-up visit, and the toothbrushing frequency were recorded. Oral hygiene status was assessed using the Simplified Oral Hygiene Index (OHI-S)^40^. OHI-S is the sum of the plaque index and calculus index, which quantify the plaque and calculus on six reference teeth (four posterior and two anterior). Plaque and calculus are scored on scales from 0 to 3, and the sum of the values is divided by the number of observed surfaces. The plaque and calculus indices are interpreted as good (0–0.6), fair (0.7–1.8), and poor (1.9–3.0). The OHI-S is interpreted as good (0–1.2), fair (1.3–3.0), and poor (3.1–6.0).

The periodontal assessment included the Community Periodontal Index for Treatment Needs (CPITN) developed and validated by the World Health Organization (WHO)^41^. A WHO basic periodontal examination probe was used. This is a lightweight metallic probe with a 0.5-mm ball tip, a black band between 3.5 and 5.5 mm, and rings 8.5 and 11.5 mm from the ball tip. The following categories were used to record periodontal status: healthy periodontium; bleeding during or after periodontal probing; presence of calculus; periodontal pocket 4–5 mm; and periodontal pocket > 6 mm.

All patients received personalized oral hygiene advice based on their clinical examination findings, as part of routine care, without any standardized intervention.

### Microbiological data

Subgingival dental plaque was collected from each participant by sampling two or three of the deepest periodontal pockets among the six aforementioned reference teeth with sterile paper points, using the PERIO-ANALYSE sampling kit (Institut Clinident, Aix-en-Provence, France). Total bacterial counts were determined by the Institut Clinident using real-time quantitative PCR, including specific counts for nine bacterial species: *Aggregatibacter actinomycetemcomitans*, *Porphyromonas gingivalis*, *Tannerella forsythia*, *Treponema denticola*, *Prevotella intermedia*, *Parvimonas micra*, *Fusobacterium nucleatum*, *Campylobacter rectus*, and *Eikenella corrodens.* In addition to total bacterial quantification, the analyses by the Institut Clinident included pathogenicity thresholds initially defined by the manufacturer for the use of antibiotic therapy.

### Statistical analyses

Analyses were performed with R (v 4.2.1); the level of statistical significance was set at *p* < 0.05^42^. Quantitative variables were described as means, standard deviations, medians, first and third quartiles, and ranges. Qualitative variables were described as frequencies and percentages. Bacterial counts and pathogenicity thresholds were transformed using the natural logarithm. Paired differences in LEDD between baseline and follow-up were assessed using the Wilcoxon signed-rank test.

For the primary objective of this study, linear mixed-effects models were used to describe the microbiological evolution during follow-up. For each bacterial species, the effect of time was estimated using a model including time as a fixed effect and participant as a random intercept, with adjustment for the individual follow-up delay. Residual normality, homoscedasticity and random‑effects distribution were verified. Effect estimates are reported on the log scale with their 95% confidence intervals (CI) and were back-transformed to express the corresponding percentage change in CFU/mL. To account for multiple testing across the ten bacterial outcomes, p-values were corrected using the Benjamini-Hochberg false discovery rate procedure.

As secondary analyses, mixed-effects logistic regression was used to estimate the within-participant change (follow-up vs. baseline) in the odds of being above the pathogenicity threshold. Models included time as a fixed effect, a participant-level random intercept, and adjustment for follow-up delay. When the mixed‑effects specification was not supported by the data, the analysis was performed using standard logistic regression. Odds ratios and their 95% CI were reported, and p-values were corrected using the Benjamini-Hochberg false discovery rate procedure.

Finally, for the secondary objective of this study, changes in periodontal clinical status between baseline and follow‑up were assessed using paired comparisons with McNemar’s chi‑squared test with continuity correction.

## Data Availability

All data generated or analyzed during this study are included in this article. Additional information can be obtained from the corresponding author upon reasonable request.

## Abbreviations

MDS-UPDRS: Movement disorder society–unified parkinson’s disease rating scale
LEDD: Levodopa equivalent daily dose
CFU: Colony-forming unit
CPP: Comité de protection des personnes
SD: Standard deviation
PD: Parkinson’s disease
PPD: Probing pocket depth
OHI-S: Simplified oral hygiene index
CPITN: Community periodontal index for treatment needs
WHO: World health organization
PCR: Polymerase chain reaction
Ln: natural logarithm
*A. actinomycetemcomitans*: *Aggregatibacter actinomycetemcomitans*
*P. gingivalis*: *Porphyromonas gingivalis*
*T. forsythia*: *Tannerella forsythia*
*T. denticola*: *Treponema denticola*
*P. intermedia*: *Prevotella intermedia*
*P. micra*: *Parvimonas micra*
*F. nucleatum*: *Fusobacterium nucleatum*
*C. rectus*: *Campylobacter rectus*
*E. corrodens*: *Eikenella corrodens*
